# Human resource primacy, dispositional optimism, and chest pain: A prospective, cross-lagged study of work, personality, and health

**DOI:** 10.1371/journal.pone.0215719

**Published:** 2019-04-24

**Authors:** Jan Olav Christensen, Morten Birkeland Nielsen, Live Bakke Finne, Stein Knardahl

**Affiliations:** Department of Work Psychology and Physiology, National Institute of Occupational Health, Oslo, Norway; Universitat de Valencia, SPAIN

## Abstract

Chest pain (CP) is common, frightening, and often medically unexplained. Occupational psychological factors are associated with somatic pain. Personality may influence both perceived working conditions and somatic health, thereby confounding associations of work with health. Despite this, very few studies have investigated the interplay between work factors, personality and pain. The current study assessed relationships of a relatively novel work factor, *human resource primacy* (HRP), and a personality factor known to be relevant to health, *dispositional optimism* (Opt), with CP across two years (N = 6714). A series of structural equation models (SEMs) were fitted, modeling “substantive” and “confounded” relationships of psychological factors with CP. A “common latent factor” (CLF) was included to account for bias by unmeasured factors that may have influenced all variables (e.g. reporting bias) and the role of optimism as a possible confounder of the relationship between HRP and CP was investigated specifically. Independent effects of HRP and Opt on CP were observed. No effects of HRP/CP on Opt were observed. Opt appeared to confound the relationship between HRP and CP to some extent. However, best fit was observed for a “reciprocal” model with independent lagged effects from HRP/Opt to CP as well as from CP/Opt to HRP. Thus, results suggested a mutual causal dynamic between HRP and CP along with an influence of Opt on both HRP and CP—implying that working conditions influence the experience of chest pain while the chest pain also influences the experience of working conditions. Optimistic dispositions may influence the experience of both work and pain, but not to an extent that fully explains their relationship. Hence, the notion that associations of HRP with CP are mere artifacts of optimistic/pessimistic reporting was not supported. More likely, complex reciprocal relationships exist between these factors, in which mutual reinforcements occur and both vicious and virtuous cycles may result.

## Introduction

Being a well-known symptom of cardiovascular disease (CVD), chest pain (CP) can be extremely disturbing. Acute CP motivates many emergency distress calls and incurs significant economic and human costs by being common and requiring extensive investigation [1]. However, it is not a specific symptom and most admitted patients are not diagnosed with CVD [2]. The majority of cases that reach the healthcare system remain unexplained or are due to conditions that are less alarming than CVD, such as gastritis, gastro-oesophageal reflux, sprains, panic disorder, etc. [3]. A recent population-representative US study reported that only 5.5% of emergency department visits for CP led to diagnoses that are considered life-threatening [4]. However, even if medical examinations do not give cause for alarm the symptom often continues to be profoundly alarming to the sufferer [5]. Nevertheless, due to the seriousness of CVD the clinical priority is typically exclusion of cardiac causes rather than positive management of risk factors and symptoms for persons presenting with CP [5].

Perception and interpretation of social contexts can profoundly influence pain problems [6]. Hence, in order to improve the management of CP in the population we may want to consider how significant contexts such as the work environment contribute to suppressing or raising the “alarm” that CP sufferers experience. Some studies have reported associations of unexplained CP with “perceived stress at work” [7, 8], but this concept is hard to distinguish from distress and not informative about modifiable aspects of work. Individual psychological factors such as depression and anxiety are established predictors of CP [1, 9]. Such knowledge is crucial in understanding pain problems, and should guide interventions and efforts to design systems and legislation that promote positive health. However, it does in a certain sense also carry limited practical information about what to modify in order to attain such results.

While psychological factors are typically investigated as predictors of CP after exclusion of cardiac causes, there is little reason to assume that such factors do not also affect patients suffering “well-defined” health problems. For instance, a 2003 review of placebo-controlled cancer treatments indicated that cancer pain was alleviated by placebo [10]. Moreover, as studies have demonstrated links of cardiovascular health with both optimism (measured by the LOT-R) [11] and psychological work factors [12], the possibiliy remains that any associations between HRP and CP could be partly attributable to impaired cardiovascular health. Hence, psychological factors could be relevant to the well-being of employees irrespective of “objective” (cardiovascular) disease and the current rationale for HRP as a potential contributor to CP could be valid any type of chest pain.

Most evidence regarding occupational psychological factors and pain pertains to a few factors, typically job demands, -control, and -support of the “Job strain model” [13, 14]. Hence, adding to the understanding of the impact of occupational psychological factors, the present study elucidated the role of *human resource primacy* (HRP) [15, 16], a factor that is long-established in organizational psychology but has been rarely studied with somatic health. HRP is a specific facet of organizational *climate* [16, 17]. Climate denotes organizational actors’ perceived trust, support, innovation, recognition and fairness [18]. *Litwin* created an early model of organizational climate [19] and proposed three primary outcomes—motivation arousal, organizational performance, and employee health. Taylor and Bowers [16] defined five dimensions of organizational climate; “decision making practices”, “motivational conditions”, “communication flow”, “technological readiness”, and “human resource primacy”. HRP was described as “the extent to which the climate, as reflected in the organization’s practices, is one which asserts that people are among the organization’s most important assets” ([20], p. 18).

Organizational climate is related to organizational *culture*. However, while culture involves long-term acquired shared views and tacit assumptions, climate is the manifestation of culture into an explicit, temporary “state of the organization” [18]. Relatively malleable, climate can be influenced by changes in systems, structures and managerial behaviors and stabilized by enduring group values and norms [21]. Hence, an important implication is that climates can be intentionally influenced by organizational practicians to obtain desirable outcomes. With regards to HRP, top-level managers may, for instance, want to consciously convey the message that employee health and well-being is a prioritized concern. According to Lazarus and Folkman an important moderator of the outcomes of psychological challenge is the way in which the challenge is appraised [22]. The organizational context may influence *secondary* appraisals which involve evaluations of available resources to deal with a challenge, such as for instance a health problem. Organizational contexts characterized by an expressed concern for employee welfare may convey the notion that the organization will provide support and assistance with problem-solving. Social contexts may influence pain by inducing expectations of negative or positive outcomes [6, 23]. Thus, it seems likely that “high-HRP-organizations” promote appraisals that may reduce the likelihood and severity of pain problems.

Work, health, and associations between work factors and health may be influenced by *personality* characteristics of employees. Personality refers to relatively enduring styles of thinking, feeling, and acting [24] and may be relevant to health by shaping ways in which individuals appraise events, challenges, and somatic sensations [25, 26]. *Dispositional optimism*, the propensity toward positive expectations and appraisals, is a personality trait that may influence pain since optimists tend to favor adaptive, problem-focused coping over catastrophization, avoidance, or denial [27]. An optimistic inclination may influence the way in which contextual factors are interpreted and thereby affect pain (nocebo/placebo) [6]. Optimists also tend to be flexible in employing relevant coping strategies [27]. Induced optimism has been found to influence pain directly [28], and although *negative* affect(ivity) may perhaps more often be considered relevant to health, positive affect(ivity) has been suggested to exert a stronger influence on health [29].

Personality may also influence work factors. For instance, studies have documented associations of personality with occupational choices and job strain [30, 31]. Hence, optimism may be thought to influence the report of both pain and work, either by bias or substantive influences. For instance, a “rosy perception mechanism” [32] may cause employees to assume the organization looks out for them and a “rosy prospection mechanism” [33] may cause them to form optimistic expectations about future health that ultimately reduce the risk of pain. This could induce a prospective association of HRP with pain which could be spurious in at least two ways. Firstly, by biasing reports of both work and pain, i.e. the reports of HRP and CP are similarly inflated. As an example, optimists tend to defer unpleasant thoughts and may ignore recent pain episodes and negative work events [34]. Secondly, by substantive effects of optimism on work and pain, but not of work on pain. This would be “substantive confounding” and not a problem of measurement, but rather of erroneously inferring causality to the association. In the latter case, the research may fall into a ‘triviality trap’ by failing to provide information about factors that can be targeted for intervention.

While *negative* affect is most often considered the potential “nuisance factor” [35], *positive* affect may play an even more prominent role. Negative thinkers may assess negative information quite realistically while optimists may discount it, ignore minor criticisms, and shy away from negative thoughts [34]. However, positive biases have rarely been taken into account, and negative affect variables have most often been treated as control variables, which contributes little towards the understanding of how personality, working conditions, and health interact [34, 36]. Generally, it has not been the norm to assess alternative or supplementary explanatory models for the observed association between psychological work factors and health, in spite of the many possible mechanisms that have been suggested [32]. Indicative of this, Lang and coworkers (2012) [37] meta-analyzed lagged effects of psychological work factors on musculoskeletal problems, intending also to report “reverse effects” of pain on work. However, none of the included studies reported such estimates.

The current study utilized two-wave full panel data to compare different models based on different assumptions regarding the links between HRP, Opt, and CP. There are reasons to suspect both confounding and substantive relationships, and these may co-exist. Based on the above, effects were expected in both directions between HRP and CP, and from Opt to CP and HRP. No longitudinal effect was expected from HRP and CP to Opt, since Opt should reflect a stable personality disposition not substantially affected by “normal” job circumstances. Thus, the following specific hypotheses were stated:

H1: Opt and HRP → CP. Dispositional optimism and human resource primacy were expected to predict chest pain.
H2: CP and Opt → HRP. Chest pain and dispositional optimism were expected to predict levels of human resource primacy.

## Materials and methods

### Procedure

The current study was based on two waves of data collected with a follow-up period of two years (mean and median time lag 23 months, range 17-36 months). Data were gathered by web-based questionnaires in the form of work environment surveys, taking place from 2004 to 2012 (baseline) and 2006 to 2014 (follow-up). Responses were treated confidentially in accordance with a license given by the Norwegian Data Inspectorate, and companies received reports of results as well as presentations prior to the study to communicate study aims and inform of the confidential treatment of information. Participation was voluntary after informed consent and the project was approved by The Regional Committees for Medical and Health Research Ethics (REK) in Norway.

### Participants

At the time of the current analyses, a total of 14398 employees had been invited to participate at both T1 and T2. Of these, 9482 (65.9%) volunteered relevant information at T1 and 6759 (71.3% of the initial sample) also at T2. The current analyses were based on the WLSMV estimator, which allows missing information for dependent variables, but not for independent variables. Thus, 45 subjects were excluded due to missing background information. Hence, the effective sample size was 6714 (70.8% of T1 responders, 46.6% of employees invited to participate at both T1 and T2) (see Table 1).

**Table 1:** Baseline descriptives for the study sample (N = 6714).

		N	%
**Age**	Mean	44.5	-
	SD	10.1	-
	Range	19–68	-
**Sex**	Male	3007	44.8
	Female	3707	55.2
**Skill level**	> 15 years	1842	27.4
	13-15 years	1623	24.2
	10-12 years	2429	36.2
	< 10 years	63	0.9
	“Unspecified”	757	11.3
**Human resource primacy**			
“Are you rewarded for a job well done	“Very little/not at all”	2336	36.2
in your company/organization	“Rather little”	1427	22.1
(money, encouragement)?”	“Somewhat”	1635	25.3
	“Rather much”	846	13.1
	“Very much”	211	3.3
“Are the employees well looked after in	“Very little/not at all”	241	3.7
your company/organization?”	“Rather little”	671	10.4
	“Somewhat”	1761	27.3
	“Rather much”	2723	42.3
	“Very much”	1045	16.2
“To what extent is management	“Very little/not at all”	363	5.7
in your company/organization concerned	“Rather little”	838	13.1
with the health and well-being of employees?”	“Somewhat”	1863	29.0
	“Rather much”	2323	36.2
	“Very much”	1031	16.1
**Dispositional optimism**			
“In uncertain times, I usually expect the best”	“Strongly disagree”	57	0.9
	“Disagree”	568	8.7
	“Neutral”	2443	37.6
	“Agree”	2919	44.9
	“Strongly agree”	509	7.8
“I hardly ever expect things	“Strongly disagree”	920	14.2
to go my way” (reversed)	“Disagree”	2462	37.9
	“Neutral”	2304	35.5
	“Agree”	718	11.1
	“Strongly agree”	88	1.4
“Overall, I expect more good things	“Strongly disagree”	92	1.4
to happen to me than bad”	“Disagree”	399	6.1
	“Neutral”	1723	26.5
	“Agree”	3547	54.6
	“Strongly agree”	731	11.3
**Chest pain at T1**	None	5969	91.7
	Light	441	6.8
	Moderate	78	1.2
	Severe	19	0.3

### Measures

#### Chest pain

The outcome measure was obtained from a previously published symptom checklist encompassing several health complaints [38]. Intensities of 21 different health complaints were assessed by asking “have you been bothered by (e.g. chest pain) the last 4 weeks?”, with optional answers “not bothered” (1), “a little bothered” (2), “rather intensely bothered” (3), and “very intensely bothered” (4). In Norwegian language the phrase “bothered by” is a common way of expressing that one has experienced a symptom. Employing a single-item measure in the current study is as a limitation, since such measures are generally considered psychometrically inferior to multiple-item measures. However, they have some beneficial properties. Most obviously, they decrease the burden on informants by reducing the number of questions pertaining to the same construct. Avoiding what informants may perceive as redundancy and repetition should increase motivation and prevent resentment [39]. Moreover, multiple-item measures risk criterion-contamination, i.e. the inclusion of construct-irrelevant items that introduce disturbances rather than construct-relevant variance [39]. Previous research has found single-item verbal pain rating scales to exhibit adequate reliability and validity, e.g. for leg- and back pain [40], and in patients with rheumatoid arthritis [41].

#### Human resource primacy

Human resource primacy (HRP) was measured with The General Nordic Questionnaire for Psychological and Social Factors at Work (QPS_Nordic_) [15]. The QPS_Nordic_ is a thoroughly validated instrument originally developed after an initiative by the Nordic Council of Ministers to address the need for a comprehensive instrument comprising constructs central to occupational health psychology research and practice.

The HRP scale of the QPS_Nordic_ consists of three items, covering aspects of *reward*, *care*, and *management involvement* in the health and well-being of employees. Specific wordings are: “Are you rewarded for a job well done in your company/organization (money, encouragement)?”, “Are the employees well looked after in your company/organization?”, and “To what extent is management in your company/organization concerned with health and well-being of employees?”. Responses were recorded with the following optional answers: 1 = “Very little or not at all”, 2 = “Rather little”, 3 = “Somewhat”, 4 = “Rather much”, and 5 = “Very much”. Response ratings reflecting agreement or satisfaction are avoided in order to minimize the impact of affective traits that may influence ratings of emotive content [15].

The ordinal Chronbach’s *α* [42], based on polychoric correlations since the items were ordered categorical and skewed, was calculated to 0.82.

#### Dispositional optimism

Dispositional optimism, the generalized expectation of positive rather than negative outcomes, was measured with three items of the “Revised Life Orientation Test (LOT-R)” [43]. Specific wordings were: “In uncertain times, I usually expect the best”, “I hardly ever expect things to go my way” (reversed), and “Overall, I expect more good things to happen to me than bad”. Response categories were 1 = “Strongly disagree”, 2 = “Disagree”, 3 = “Neutral”, 4 = “Agree”, and 5 = “Strongly agree”. The use of agreement rating is in this case desirable in order to tap a global affective trait.

The ordinal Chronbach’s *α* was calculated to 0.67. This is below the often cited (although arbitrary) lower limit of 0.7 [42]. However, since *α* is a function of the number of items and may be underestimated with less than ten items, inter-item correlations can be calculated to confirm reliability when few items are employed [44]. The inter-item correlation for the optimism scale was 0.4, which is above the cutoff of 0.15 suggested for items that tap the same construct [44].

#### Covariates

Dependent variables were regressed on age, sex, and “skill level”. “Skill levels” were determined according to the International Standard for Classification of Education (ISCED), reflecting typical educational requirements of different occupations. This classification was based on a Norwegian adaptation of the International Standard Classification of Occupation (ISCO-88) by Statistics Norway, reflecting occupations typically requiring education equivalent to (1) a postgraduate university degree (>15 years), (2) 1-3 years at university or college (13-15 years), (3) 1-3 years of secondary education (10-12 years), (4) no more than 9 years of primary education, and (5) unspecified (occupations for which required education may vary substantially).

### Statistical analyses

Analyses were conducted with Mplus Version 7.11 [45]. The level of statistical significance was set to p<0.05.

Structural equation modeling (SEM) with latent and manifest variables was utilized for model testing. Latent variables are non-observable variables, such as personality dispositions, that can be *inferred* by employing factor analysis to extract shared variance of multiple items [46]. This is based on the assumption that variation in a set of observed items is caused by (1) a common, “latent” factor (e.g. “optimism”), (2) systematic, item-unique “residual” influences, i.e. extraneous sources of variance influencing the response to items independently of the factor of interest, and (3) random error. The factor analysis extracts the reliable part and corrects for measurement error. Latent variables can then be included in a set of simultaneously specified “structural equations” to model their relationships with each other. A robust weighted least squares estimator (WLSMV) was employed to fit the structural equation models, which is appropriate with ordered categorical, non-normally distributed items [45].

Model fit was judged by the comparative fit index (CFI; values >0.95 indicate good fit [46]), the root mean square error of approximation (RMSEA; values <0.06 indicate good fit [46]), and the Tucker-Lewis index (TLI; values >0.96 indicate good fit [46]).

*Probit* regressions were employed for observed, categorical dependents (in this case; CP) and *linear* regressions for latent, continuous dependent variables (HRP and Opt). Probit coefficients express *the effect of a unit change of the predictor on the z-score of the outcome*. The effect on the *probability* of a level of the outcome is, however, contingent on the specific levels of all predictors in the model. Hence, the interpretation of probit regression coefficients in terms of effect size is not straightforward and not necessarily comparable for different outcomes and covariates. Therefore, interpretations typically consider direction and statistical significance rather than magnitudes of effect. In order to express the effect of exposures on chest pain in a way that captures the practical significance, predicted probabilities of chest pain occurring were plotted as functions of HRP and optimism.

Since the sample was recruited at the organizational level, a sandwich estimator [45] was obtained to correct standard errors for non-independence of observations clustered within companies.

*Cross-lagged analysis* [47] involves modeling bidirectional effects across time—in this case, from T1 psychological factors to T2 health and from T1 health to T2 psychological factors—so that competing models can be statistically compared. Although establishing *the existence* of causal effects is beyond the scope of observational studies, cross-lagged analyses enable comparison of models derived from different causal assumptions. Some of these models are *nested*, i.e. the more complex models can be specified by adding effect paths to the more parsimonious models. The relative tenability of nested models can be judged by determining whether adding effect paths improves model fit to an extent that justifies the loss of parsimony [47].

**Fig 1:**
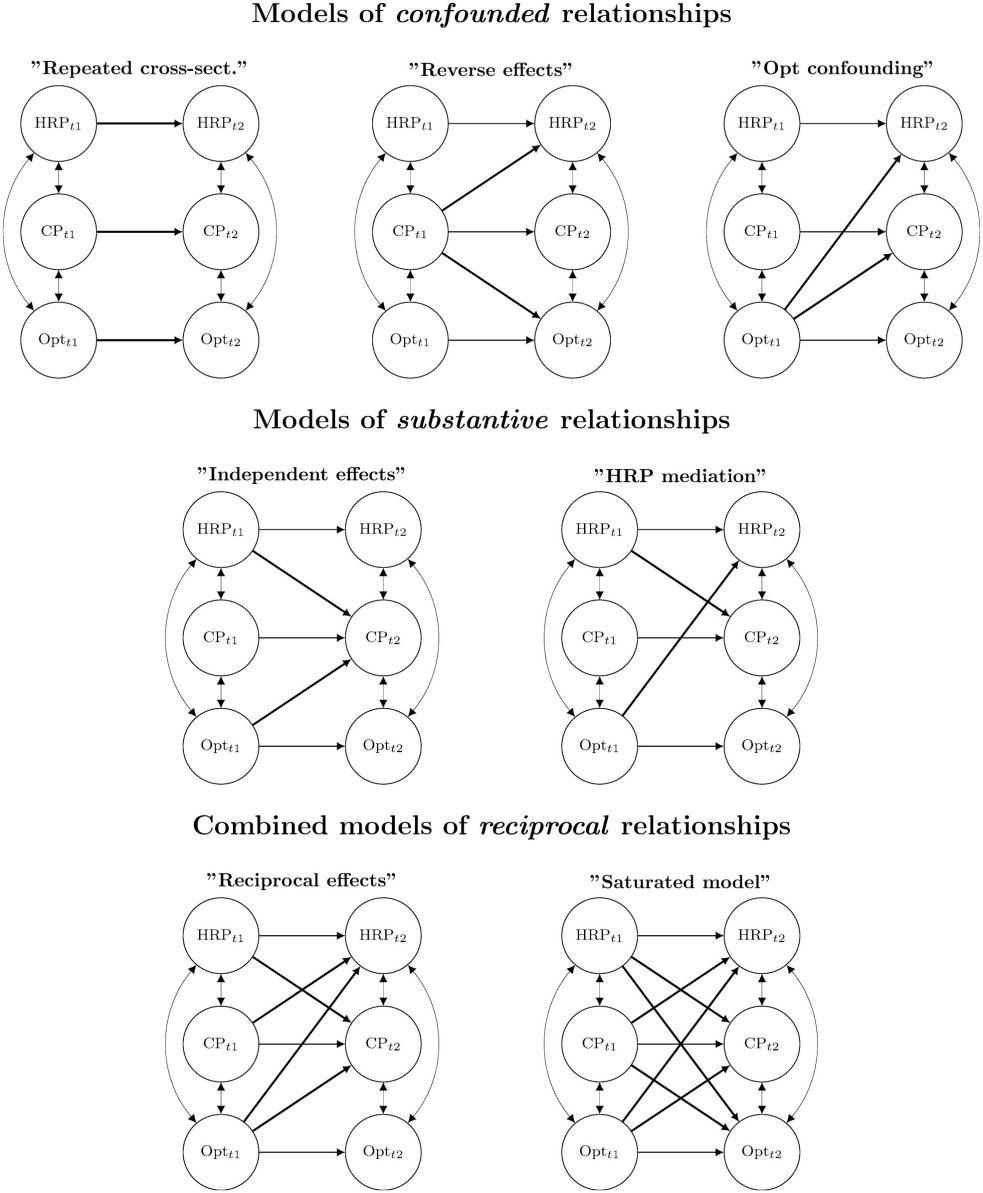
Illustration of structural equation models specifying relationships of human resource primacy (HRP) and dispositional optimism (Opt) with chest pain (CP). Sex, age, skill level, observed indicators, and the single common latent factor (CLF) are omitted from the figure.

Several models were specified, reflecting assumptions of both *confounded* and *substantive* relationships between psychological factors and pain (see Fig 1). All models included a “common latent factor” (CLF), a single factor defined by all observed items of HRP, Opt, and CP. This factor was modeled as uncorrelated with HRP and Opt and factor loadings from the CLF to each observed item were constrained to equality. This reflects an assumption that one single underlying factor influenced the report of all items equivalently. This strategy has been recommended by Podsakoff and coworkers [48] to correct for method bias.

The basic prospective model included no cross-lagged effects. Since this implies that any prospective relationship of psychological factors with pain would result from cross-sectional associations carried forward in time through the stability of the involved factors, this model was denoted *”repeated cross-sectional”*. It is worth noting that cross-sectional associations could reflect short-term causal effects, although such causality remains obscure under the current modeling framework. All cross-lagged models were specified by adding effect paths to the “repeated cross-sectional” model. Thus, two “confounding models” were specified; a “reverse effects” model with effects from CP T1 to Opt/HRP T2 and a model of “confounding” effects from Opt T1 to HRP/CP T2 (see Fig 1). Cross-lagged “substantive models” reflected situations in which (1) OPT and HRP exert *independent* effects on CP across time, or (2) individual disposition (Opt) affects work/perceptions of work, which in turn affects health (”HRP mediation”) (see Fig 1). A “*reciprocal effects*” model was estimated, combining all the previous models by specifying effect paths from HRP at T1 to CP at T2, from Opt at T1 to CP and HRP at T2, and from CP at T1 to HRP at T2 (Fig 1). Finally, a “saturated” reciprocal effects model was specified, including paths also from HRP and CP at T1 to Opt to T2. This was done for the purpose of model comparison, as HRP and CP were not expected to predict Opt.

Thus, the “repeated cross-sectional model” was nested in all other models, which were all nested in the “saturated” model. Nested models were compared by robust chi-square difference tests (the DIFFTEST option of Mplus).

Each single item was correlated with itself across time, modeling the stability of residual influences on observed items not due to the estimated latent factors [47]. For all models residual variances of dependent variables were correlated, providing estimates of associations between outcomes *after* lagged effects. If effects occurred within a shorter time frame than the study period, this may be captured by residual covariances.

## Results

### Preliminary analyses

Prior to conducting structural equation modeling, measurement models of the latent constructs (HRP and Opt) were specified and tested by confirmatory factor analyses (CFA). First, a CFA for both factors was conducted with cross-sectional data from the first measurement point. Results indicated good fit (RMSEA 0.053, 90% confidence interval 0.046-0.060; CFI 0.995; TLI 0.990) (analyses not shown).

For lagged models to reflect information about processes unfolding over time measures must represent the same factors at each time point—different scores at different occasions should reflect change rather than unreliable measurement. The latent variable approach enables testing the assumption of *longitudinal measurement invariance* [47]. A series of models were run with HRP and Opt at both time points, in accordance with Little et al. [47], to reflect (1) configural invariance; i.e. identical patterns of factor loadings, (2) loading invariance; i.e. loadings of each item on corresponding factors constrained to equality across time points, and (3) threshold/intercept invariance; i.e. both factor loadings and thresholds/intercepts of the factor indicators constrained to equality. All of these models exhibited excellent fit (configural invariance model: RMSEA 0.034, 90% CI 0.031-0.037; CFI 0.992, TLI 0.987; loading invariance model: RMSEA 0.031, 90% CI 0.028-0.034; CFI 0.992; TLI 0.989, threshold invariance model: RMSEA 0.027, 90% CI 0.024-0.029; CFI 0.991; TLI 0.992) (analyses not shown). A difference in the CFI of more than 0.01 upon introduction of constraints has been proposed to support rejection of the hypothesis of factorial invariance [49]. Furthermore, the threshold invariance model exhibited the lowest RMSEA, indicating that the most constrained model was tenable [47]. Full measurement invariance was thus supported, and all models were subsequently run with factor loadings and thresholds constrained to equality across time.

Nonresponse analyses were conducted to compare odds of response with odds of non-response at T1 based on sex, age, and skill level. Non-response was less likely for the “unspecified” skill level, i.e. no formal educational requirements, such as managers and politicians (OR 0.45, 95% CI 0.32-0.63) (not shown). Sex and age were not statistically significantly related to participation (ORs 0.79 and 0.99, respectively). Attrition was assessed by predicting T2 non-response among employees that responded at T1. Age, sex, skill level, HRP, Opt, and CP were entered as predictors. Dropout was more likely for employees in occupations requiring 13-15 and 10-12 years as opposed to more than 15 years of education (OR 1.61, 95% CI 1.14-2.27 and OR 1.60, 95% CI 1.21-2.10, respectively) (not shown). Sex, age, Opt, HRP, and CP at T1 were not associated with dropout.

### Self-reported diagnoses related to heart disease

The focus of the current study was on the influence of psychological factors on the experience and report of CP irrespective of potential underlying organic causes. However, some data were available in the form of self-reported diagnoses—an unstructured, open-ended questionnaire item was presented to subjects, asking them to report any disease or injury diagnosed by a medical doctor. Importantly, this item had some limitations, such as not specifying a time frame and not discriminating between non-response and not having a diagnosis. However, with these limitations in mind, a variable was constructed to reflect diagnosed heart disease by defining a search string to identify subjects reporting diagnoses containing one or more of the following strings: “angina-”, “coronary-”, “myocard-”, “heart-”, “infarct-”, “cardio-”, and “athero-”. The results were screened to confirm the relevance of the diagnoses. Based on this, 109 (1.6%) subjects reported a diagnosis at T1, 132 (2.0%) at T2, and 70 (1.0%) at both time points. By comparison, in 2012, 66029 (1.3%) patients had a diagnosis of ischaemic heart disease in the general Norwegian population [50], and in the current sample 8.3% reported chest pain at baseline. Hence, the current measure seems reasonable, bearing the aforementioned limitations in mind. Analyses were carried out both *with* and *without* subjects that reported a diagnosis, and no substantive differences in associations were observed. Therefore, in the following results from analyses of the full inclusive sample are reported.

### Cross-lagged analyses

#### Model fit and effect estimates

Zero-order correlations between HRP, Opt, and chest pain at both measurement occasions can be seen in Table 2.

**Table 2:** Zero-order correlations between human resource primacy (HRP), dispositional optimism (Opt), and chest pain (CP) at both measurement occasions.

	Opt T1	HRP T1	Opt T2	HRP T2	CP T1
Opt T1					
HRP T1	0.326				
Opt T2	0.700	0.252			
HRP T2	0.268	0.689	0.326		
CP T1	-0.202	-0.208	-0.185	-0.185	
CP T2	-0.226	-0.218	-0.232	-0.197	0.619

Note: all correlations statistically significant at the *p* < 0.01 level

All cross-lagged models exhibited excellent fit (see Table 3). Fit indices were almost identical between models—RMSEAs ranged from 0.019 (90% CI 0.017-0.021)—0.020 (90% CI 0.018-0.022), CFIs were 0.988 and TLIs were 0.987 for all models.

**Table 3:** Model fit for cross-lagged structural equation models (N = 6714).

	RMSEA (90% CI)	CFI	TLI
**Confounding models**			
“Repeated cross-sectional”	0.019 (0.017-0.021)	0.988	0.987
“Opt confounding”	0.019 (0.017-0.021)	0.988	0.987
“Reverse effects”	0.019 (0.018-0.021)	0.988	0.986
**Substantive models**			
“Independent effects”	0.019 (0.017-0.021)	0.988	0.987
“HRP mediation”	0.019 (0.017-0.021)	0.988	0.987
**Combined models**			
“Reciprocal effects”	0.019 (0.017-0.021)	0.988	0.987
“Saturated model”	0.020 (0.018-0.022)	0.988	0.987

CFI: Comparative Fit Index, TLI: Tucker Lewis Index, RMSEA: Root Mean Square Error of Approximation

Coefficients for cross-lagged effects and residual covariances of the cross-lagged models are given in Table 4.

**Table 4:** Effect estimates from cross-lagged structural equation models of relationships between human resource primacy (HRP), dispositional optimism (Opt), and chest pain (CP) (N = 6714).

Cross-lagged effects	Est	p	Residual covariances	Est	p
**“Repeated cross-sectional”**					
None specified	-	-	Opt T2 ↔ CP T2	**-0.112**	0.000
			HRP T2 ↔ CP T2	**-0.067**	0.000
			HRP T2 ↔ Opt T2	**0.071**	0.000
**“Opt confounding”**					
Opt T1 → HRP T2	**0.029**	0.004	Opt T2 ↔ CP T2	**-0.054**	0.004
Opt T1 → CP T2	**-0.218**	0.000	HRP T2 ↔ CP T2	**-0.056**	0.000
			HRP T2 ↔ Opt T2	**0.060**	0.000
**“Reverse effects”**					
CP T1 → HRP T2	-0.006	0.601	Opt T2 ↔ CP T2	**-0.118**	0.000
CP T1 → Opt T2	0.010	0.548	HRP T2 ↔ CP T2	**-0.064**	0.000
			HRP T2 ↔ Opt T2	**0.071**	0.000
**“Independent effects”**					
HRP T1 → CP T2	**-0.123**	0.009	Opt T2 ↔ CP T2	**-0.070**	0.000
Opt T1 → CP T2	**-0.161**	0.001	HRP T2 ↔ CP T2	**-0.043**	0.009
			HRP T2 ↔ Opt T2	**0.067**	0.000
**“HRP mediation”**					
Opt T1 → HRP T2	**0.031**	0.002	Opt T2 ↔ CP T2	**-0.103**	0.000
HRP T1 → CP T2	**-0.189**	0.000	HRP T2 ↔ CP T2	**-0.034**	0.038
			HRP T2 ↔ Opt T2	**0.061**	0.000
**“Reciprocal effects”**					
Opt T1 → HRP T2	**0.026**	0.015	Opt T2 ↔ CP T2	**-0.068**	0.001
CP T1 → HRP T2	**-0.026**	0.007	HRP T2 ↔ CP T2	-0.021	0.192
HRP T1 → CP T2	**-0.155**	0.001	HRP T2 ↔ Opt T2	**0.060**	0.000
Opt T1 → CP T2	**-0.151**	0.002			
**“Saturated model”**					
HRP T1 → Opt T2	0.007	0.689	Opt T2 ↔ CP T2	**-0.054**	0.011
CP T1 → Opt T2	-0.021	0.093	HRP T2 ↔ CP T2	-0.021	0.198
Opt T1 → HRP T2	**0.028**	0.010	HRP T2 ↔ Opt T2	**0.058**	0.000
CP T1 → HRP T2	**-0.026**	0.007			
HRP T1 → CP T2	**-0.152**	0.001			
Opt T1 → CP T2	**-0.164**	0.001			

All models included sex, age, skill level, and a single common latent factor (CLF) that all items are regressed on. Unstandardized effect estimates are shown, probit regression coefficients when CP is dependent, linear regression coefficients when Opt or HRP are outcomes.

In the “**repeated cross-sectional**” model stability coefficients for the three focal variables were the following: HRP, unstandardized linear regression b 0.675, p<0.001; Opt, unstandardized linear regression b 0.697, p<0.001; CP, unstandardized probit regression b 0.783, p<0.001) (not shown).

The model where Opt was specified to confound the relationship between HRP and CP exhibited statistically significant effects both from Opt T1 to HRP T2 (linear regression b: 0.029, p = 0.004) and from Opt T1 to CP T2 (probit b: -0.218, p<0.001).

No statistically significant “reverse” effects were indicated by the “**reverse effects**” model (T1 CP → T2 HRP: -0.006, p = 0.601, T1 CP → T2 Opt (0.010, p = 0.548).

The “**independent effects**” model exhibited statistically significant, mutually adjusted effects of T1 HRP on T2 CP (-0.123, p = 0.009) and T1 Opt on T2 CP (-0.161, p = 0.001).

The “**HRP mediation**” model exhibited statistically significant effect paths from T1 Opt to T2 HRP (-0.031, p = 0.002) as well as from T1 HRP to T2 CP (-0.189, p<0.001).

The “**reciprocal effects**” model exhibited statistically significant effect paths from T1 Opt to both T2 HRP and T2 CP (0.026, p = 0.015 and -0.151, p = 0.002, respectively), from T1 CP to T2 HRP (-0.026, p = 0.007), and from T1 HRP to T2 CP (-0.155, p = 0.001). These associations remained statistically significant in the “**saturated**” model (HRP → CP: -0.152, p = 0.001; Opt → CP: -0.164, p = 0.001) as well as from T1 Opt and T1 CP to HRP (Opt → HRP: 0.028, p = 0.010; CP → HRP: -0.026, p = 0.007). However, no effect was indicated for the added paths from T1 HRP and T1 CP to T2 Opt (HRP → Opt: 0.007, p = 0.689; CP → Opt: -0.021, p = 0.093). Moreover, significant loadings from CLF to all observed items suggested the additional existence of an underlying factor that influenced all items (probit coefficient 0.24, p<0.001, not shown).

Residual covariances between HRP, Opt, and CP at T2 were statistically significant for most models, indicating that the specified lagged effects did not fully account for associations between variables (see Table 4). The only exceptions were the “**reciprocal**” and “**saturated**” models, for which the residual covariance between HRP and CP were not statistically significant (-0.021, p = 0.192 and -0.021, p = 0.198, respectively).

#### Comparison of nested models

The chi-square difference tests favored the more complex cross-lagged model in all comparisons with two excepttions—the *reverse effects* model did *not* represent an improvement over the repeated cross-sectional model (Δ*χ*^2^ 1.191, df 2, p = 0.551) (Table 5) and the saturated model did *not* represent an improvement over the/reciprocal effects/ model (Δ*χ*^2^ 2.176, df 2, p = 0.337) (Table 5). Hence, there was no support for the inclusion of “reverse effects” from CP or HRP to Opt.

**Table 5:** Chi-square difference tests comparing nested structural equation models (N = 6714).

	Comparisons with “repeated cross-sectional”		Comparisons with “reciprocal”		Comparisons with “saturated”	
	Δ*χ*	p	Δ*χ*	p	Δ*χ*	p
**Confounding models**						
“Repeated cross-sectional”	ref	-	46.017	0.000	51.370	0.000
“Opt confounding”	34.684	0.000	13.141	0.001	16.559	0.002
“Reverse effects”	1.191	0.551	not nested	-	50.911	0.000
**Substantive models**						
“Independent effects”	30.044	0.000	15.386	0.001	17.995	0.001
“HRP mediation”	27.655	0.000	18.776	0.000	21.057	0.000
**Combined models**						
“Reciprocal effects”	46.017	0.000	ref	-	2.176	0.337
“Saturated model”	51.370	0.000	2.176	0.337	ref	-

A statistically significant chi-square difference test indicates that the more complex model is preferred.

Statistically, then, the preferred model was the “**reciprocal effects**” model, specifying “normal effects” from Opt to both HRP and CP and from HRP to CP, and a “reverse effect” from CP to HRP, but no “reverse” effects from CP or HRP to Opt.

### Practical significance: Model-predicted probabilities of new-onset chest pain

**Fig 2:**
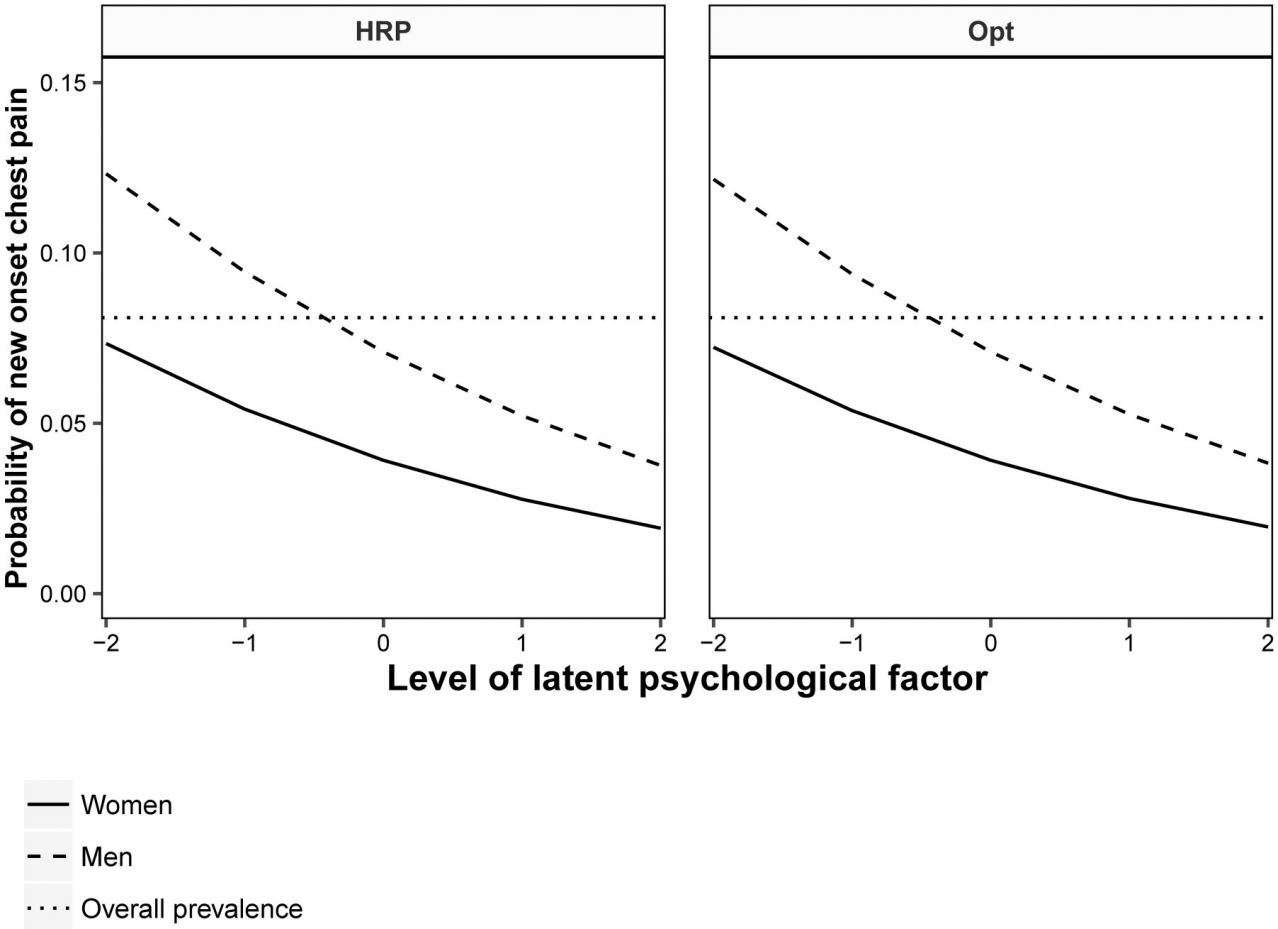
Predicted probability of new-onset chest pain at T2. Probabilities are estimated by levels of *human resource primacy* (HRP) and *dispositional optimism* (Opt) at T1 for a 45-year old employee with 10-12 years of education, a low score on the general common latent factor (CLF), and an average level of the other psychological factor.

Since probit effect estimates are not readily interpretable, predicted probabilities of chest pain were plotted as functions of HRP and optimism to demonstrate the difference in risk between subjects scoring different levels of these variables. Probabilities of T2 CP were plotted based on the final “reciprocal effects” model. The probability of T2 CP occurring was plotted as a function of HRP and Opt (Fig 2) conditional on specific values of covariates. Thus, the probability of CP for different levels of HRP and Opt (as latent variables ranging -2 to 2) were given for men and women that were pain-free at T1, with age set to the sample mean of 45, for employees with 10-12 years of education, with a low score on the CLF (e.g. employees with a low propensity toward a set response style), and mean levels of the other psychological factor (HRP or Opt).

## Discussion

The current results suggested that employee chest pain can be influenced by companies’ demonstrated concern for employee health and well-being. Employee dispositions toward optimistic thinking are also likely to influence CP, both independently of HRP and by influencing the perception of working conditions. The results also suggested that optimists tend to report high HRP with low CP, and that a general unmeasured third factor (CLF) influenced the report of all items. This supports a notion of confounding by personality and other unknown influences on subjective report. However, such influences did not seem to fully explain the associations between the psychological work factor and pain. The residual T2 association between HRP and CP was statistically significant in the “Opt confounding” model, but not in the “reciprocal” model, with HRP T1 included as predictor (see Table 4). The statistical tenability of the reciprocal model suggested that adding HRP as a predictor of CP improved prediction compared to the model in which Opt was the sole predictor. Thus, the results suggested that work, optimism, and chest pain are linked together in several ways, some of which should be amenable to modification.

The “reciprocal model” suggested Opt influences HRP, which in turn affects CP. Spector [51] has argued that dispositions may play substantive rather than confounding roles in “stressor-strain” relationships. For instance, effects of negative affectivity (NA) on psychological health have been found to be partially mediated by “work hassles”. Girardi and coworkers [52] observed associations of NA with proinflammatory cytokines (IL-1*β*, IL-12, IL-17) which were fully mediated by interpersonal work conflicts. Importantly, although dispositions themselves are unlikely targets of workplace interventions, modifying their aversive consequences (e.g. conflict or low HRP) should be salutogenic.

The “reverse effects” model was the least tenable model. In fact, the lack of prospective association of HRP and CP with subsequent Opt demonstrated the divergent validity of the Opt measure—personality should not be substantially affected by work and health. However, one would expect HRP and CP to predict Opt if self-report methods suffer automatically and to a critical extent from the influence of informant traits. Instead, the more likely explanation seems to be that these factors measure different constructs, meaningfully reflected by incumbents’ reports, rather than merely tapping an unspecific negative-positive continuum.

While much research has documented the impact of organizational climate on individual and group performance [21] less is known about effects on health. However, it has been associated with musculoskeletal complaints [53], and HRP in specific has been associated with work ability [54], mental distress [55], job satisfaction [56], and role conflict [57]. A similar concept to HRP, “psychosocial safety climate”—pertaining to policies, practices, and procedures to protect employee *psychological* health [58]—was recently found to predict working conditions that in turn predicted musculoskeletal pain [59].

One limitation of the current study that should be kept in mind when interpreting the results is the employment of a single item measure of chest pain, which means that the phrasing of the item may have a larger impact on the report of pain than if it were a part of a multi-item measure. For instance, subjects were asked whether they had been “bothered” by pain. While this is a common way of expressing symptoms in the Norwegian language, the possibility remains that some respondents reported their level of functional limitations or distress due to pain, rather than the intensity of pain itself. Hence, the current outcome measure may reflect a more general concept of “pain problem” than pain intensity.

Future studies should gain an understanding of how HRP promotes positive health. It has been linked with low levels of bullying [60], which in turn may influence symptoms of somatization as well as cortisol levels [61] and mental distress [62]. Low HRP may also imply uncertainty regarding whether ones workplace will ensure healthy working conditions. Just as empathic, patient-centered clinical interviews can increase pain thresholds [63], one may discern that an environment in which employees are convinced their health is protected promotes positive health. A general perception of being cared for and supported may also attenuate uncertainty regarding existing health concerns. Uncertainty regarding forthcoming pain-stimuli has been shown to be hyperalgesic [64]. Moreover, feeling cared for may influence *mood*. Experimentally induced positive moods, for instance, may alleviate pain by inhibiting catastrophic thoughts [65].

## Conclusion

In conclusion, although the current study should be replicated with other measures and methods, the results suggested prospective relationships of both human resource primacy and dispositional optimism with chest pain. The data did not rule out the possibility that individual and situational characteristics may bias reports of work and health and consequently the association between them. However, they suggested that a relationship exists beyond such bias and did not give reason to dismiss subjective reports of work and health by default. Instead, they suggest researchers should attempt to disentangle various sources of variance and covariance by systematically and explicitly taking them into account when specifying models to analyze self-reported data reflecting subjective constructs. HRP is a measure of employees’ appraisal of management emphasis on human resources and thus ultimately a subjective factor. Nonetheless, the current results, if replicated in further studies, should be valuable to organizational practitioners by demonstrating the significance of effectively expressing a sincere interest in employee health promotion. If this organizational imperative is effectively conveyed, the act of conveyance in itself could contribute to the improvement of health.
